# ﻿*Johnius javaensis*, a new species of croaker (Acanthuriformes, Sciaenidae) from the southern coast of Java, Indonesia

**DOI:** 10.3897/zookeys.1259.160425

**Published:** 2025-11-11

**Authors:** Bai-an Lin, John J. Pogonoski, Fahmi, Kunto Wibowo, Ning Labbish Chao, Mathew Seymour

**Affiliations:** 1 School of Biological Sciences, The University of Hong Kong, Hong Kong SAR, China The University of Hong Kong Hong Kong SAR China; 2 CSIRO Australian National Fish Collection, National Research Collections Australia, Hobart, Tasmania, Australia CSIRO Australian National Fish Collection, National Research Collections Australia, Hobart Hobart, Tasmania Australia; 3 Research Center for Oceanography, National Research and Innovation Agency, Jakarta, Indonesia Research Center for Oceanography, National Research and Innovation Agency Jakarta Indonesia; 4 Museum Zoologicum Bogoriense, Research Center for Biosystematics and Evolution, National Research and Innovation Agency, Cibinong, Indonesia Museum Zoologicum Bogoriense, Research Center for Biosystematics and Evolution, National Research and Innovation Agency Cibinong Indonesia; 5 National Museum of Marine Biology and Aquarium, and Graduate Institute of Marine Biology, National Dong Hwa University, Pingtung, Taiwan National Dong Hwa University Pingtung Taiwan

**Keywords:** Mental barbels, morphology, phylogeny, Southeast Asia

## Abstract

In this study, we describe a newly recognized croaker (Sciaenidae), *Johnius javaensis***sp. nov.** based on five specimens (87–121 mm in standard length) from the southern coast of Java, Indonesia. Specifically, taxonomic reassessment of sciaenids collected from fish markets on the islands of Java, Bali, and Lombok of Indonesia has revealed that specimens previously identified as *Johnius heterolepis* represent this new species. *Johnius javaensis***sp. nov.** is characterized by five vesicular mental barbels on the chin, 30–32 dorsal-fin rays, seven or eight scale rows below the lateral line; 11 or 12 lower gill rakers, 11 or 12 swim-bladder appendages, and ventral margin of the sagitta head expanded into a distinct convexity. Historically, four *Johnius* species (*J. amblycephalus*, *J. fuscolineatus*, *J. macropterus* and *J. mannarensis*) were reported to have one mental barbel on the chin. The new species is readily distinguished from all other 33 described *Johnius* species by possessing five vesicular mental barbels on the chin. A phylogenetic analysis of 21 *Johnius* species based on the 521-bp COI gene confirms that the new species is placed as a sister species of *J. macropterus*, based on an average of dissimilarity 8.16%.

## ﻿Introduction

There are around 300 valid species of sciaenid fishes worldwide (Fricke et al. 2025) and around 100 species of sciaenid fishes in the Indo-West Pacific (IWP) region (Trewavas 1977; Lal Mohan et al. 1984; Sasaki 1989; Chao et al. 2019). The genus *Johnius* Bloch, 1793 is the largest monophyletic group of Sciaenidae endemic to the IWP region, with 33 recognized species (Sasaki 2001, 2022; Chao et al. 2019; Geenita et al. 2025). *Johnius* was designated by Gill, 1862, and defined by Sasaki (1989) with the following distinguishing (diagnostic or shared) characters: 1) a hammer-shaped swim-bladder with 9–20 pairs of arborescent lateral appendages; 2) the first lateral branch of swim-bladder appendage extending to dorsal corner of gill opening, appearing externally on the supracleithrum bones under the skin; 3) a unique triangular sagittal otolith; and 4) an enlarge lapilli otolith.

*Johnius* species usually have a small mouth, sub-terminal to inferior in position, with four species (*Johnius amblycephalus* (Bleeker, 1855), *Johnius fuscolineatus* (von Bonde, 1923), *Johnius macropterus* (Bleeker, 1853), and *Johnius mannarensis* Lal Mohan, 1971) having one mental barbel on the chin, and one species (*Johnius elongatus* Lal Mohan, 1976) reported to have two short, irregular tags (thickened skin) between the medial and first lateral mental pores with no barbel (Lal Mohan et al. 1984; Sasaki 2022). Recent re-identification of the sciaenids in the Australian National Fish Collection (Hobart) collected from fish markets of the islands of Java, Bali, and Lombok in Indonesia have revealed an undescribed sciaenid species with five vesicular mental barbels on the chin from the southern coast of west and central Java (White et al. 2013).

Herein, we describe *J. javaensis* sp. nov., which was previously misidentified as *Johnius heterolepis* Bleeker, 1873 by White et al. (2013), but it differs from the true *J. heterolepis* in having five vesicular mental barbels on the chin (vs absent) and having more dorsal-fin rays (30–32 vs 25–28) (Sasaki 1992). The new species is genetically most similar to *J. macropterus*, from which it can be readily distinguished by possessing five vesicular mental barbels on the chin (vs one short mental barbel). Additionally, we conducted a phylogenetic analysis of 21 species of *Johnius* species using partial cytochrome c oxidase subunit I (COI) gene sequences to assess further the extent of differentiation of the new species from other congeners. An identification key to 11 *Johnius* species around the island of Java is also included in this study.

## ﻿Materials and methods

**Figure 1.**
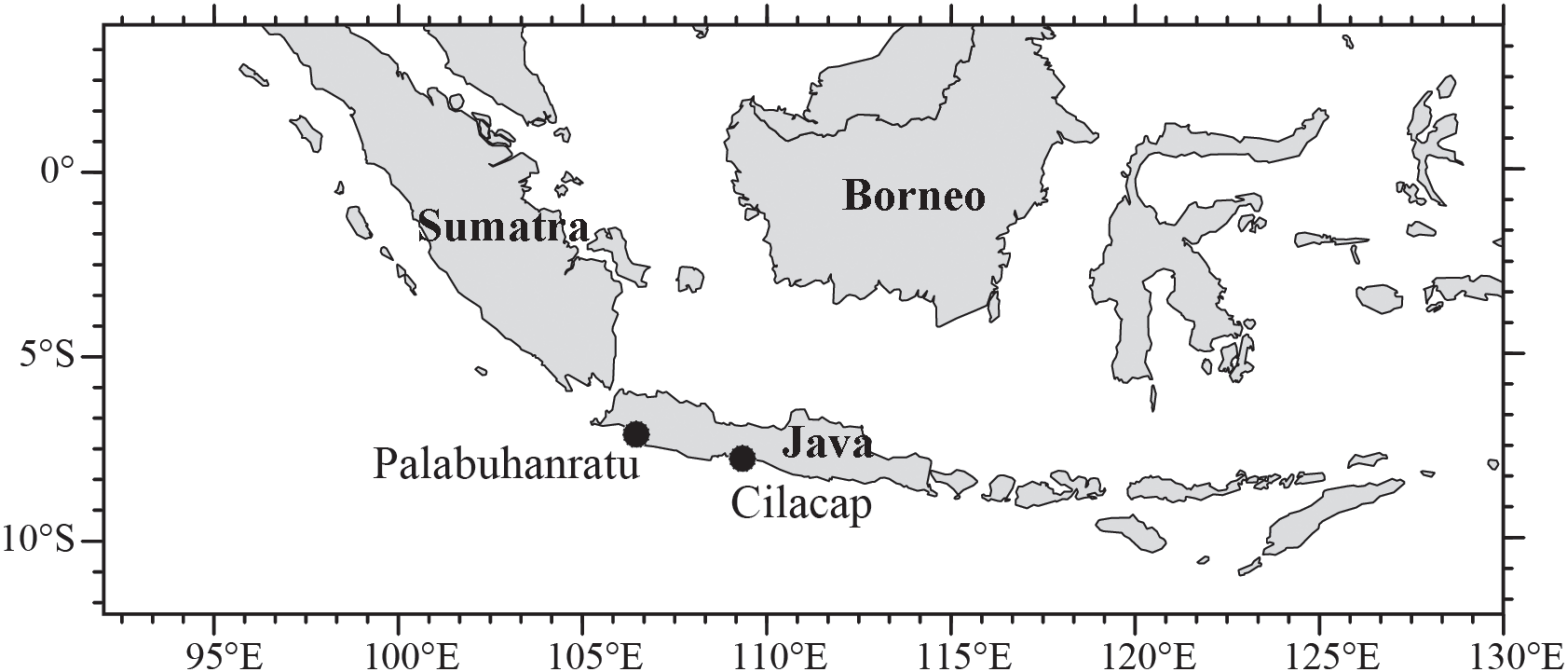
Localities of *Johnius javaensis* sp. nov. The type locality is Cilacap.

All specimens were examined at the Commonwealth Scientific & Industrial Research Organisation (**CSIRO**) Fish Collection, Hobart, Australia. The holotype and one paratype were deposited at the Museum Zoologicum Bogoriense (**MZB**), Bogor, Indonesia and the other paratypes were deposited at the CSIRO collection. All samples were collected from a joint collaborative project (ACIAR) between Indonesia and Australia by Indo-Oz project team from 2004 to 2010. The sampling locality of type specimens is shown in Fig. 1.

Counts and measurements follow Sasaki and Kailola (1991) and Chao et al. (2019). Swimbladder appendages were examined in two specimens (MZB 28379, holotype and CSIRO H 8376-12, paratype). Institutional acronyms follow Sabaj (2025). Standard length and head length are expressed as SL and HL, respectively. Counts and measurements of the paratypes were given in parentheses where values differ from the holotype. We also used descriptions and identification keys to check against the new species (Lal Mohan 1975; Trewavas 1977; Lal Mohan et al. 1984; Sasaki and Kailola 1991; Sasaki 1992, 1994, 1996, 1997, 1999, 2001, 2022; Chao et al. 2019; Hanafi et al. 2022, 2024). Two *Johnius* species, *J. macropterus* and *J. amblycephalus*, each with one mental barbel, were collected with the new species in the same region and are included as comparative materials in this study for mental barbel and morphology comparison.

### ﻿Molecular analysis

The 523-651 bp partial mitochondrial COI gene of *J. javaensis* sp. nov. (BW-A5764, BW-A5765, and BW-A9050 in BOLD and PV711386 to PV711388 in GenBank) were deposited on both the Barcode of Life Data System website (BOLD, https://boldsystems.org/) and GenBank (NCBI) by Australian National Fish Collection, CSIRO. To construct the phylogenetic relationship between *J. javaensis* sp. nov. and other *Johnius* species, the partial mitochondrial COI gene for 20 *Johnius* species were retrieved from GenBank, and all these sequences have been taxonomically verified in previous studies (Table 1). *Dendrophysa russelli* (Cuvier, 1829) was selected as the outgroup. Alignments of COI sequences were conducted by using the MAFFT (v. 7.511) online version alignment for multiple sequences (Katoh et al. 2019) with the default settings of the E-insi algorithm. The aligned sequences were 521 bp in length. The maximum-likelihood (ML) tree was inferred using MEGA 11 (Tamura et al. 2021) with 1000 bootstrap pseudo-replications. In addition, the Kimura 2-parameter distance (K2P distance) of the 521-bp COI gene was calculated between *J. javaensis* sp. nov. and other species within the genus.

**Table 1.** Taxa, vouchers, locality, and GenBank/BOLD accession numbers of *Johnius* species used in the phylogeny analysis.

Species	Voucher	Country	Locality	Accession no.	References
*J. javaensis* sp. nov.	MZB 28379	Indonesia	Cilacap	PV711386 (BW-A5765)	This study
*J. javaensis* sp. nov.	CSIRO H 7697-11	Indonesia	Cilacap	PV711387 (BW-A5764)	This study
*J. javaensis* sp. nov.	CSIRO H 8376-12	Indonesia	Cilacap	PV711388 (BW-A9050)	This study
*J. amblycephalus*	—	China	Naozhou island	MF083698	Chao et al. 2019
*J. belangerii*	—	China	Xiamen	MG917695	Chao et al. 2019
*J. borneensis*	—	China	Dongshan island	MG917696	Chao et al. 2019
*J. carouna*	—	China	Zhanjiang	MF004312	Chao et al. 2019
*J. coitor*	NMMBA 37096	Malaysia	Sibu	OK255665	Hanafi et al. 2024
*J. distinctus*	—	China	Xiamen	MF083699	Chao et al. 2019
*J. dussumieri*	—	South Africa	Tugela Banks	JF493701	Steinke et al. 2016
*J. elongatus*	—	India	−	EF534125	Lakra et al. 2009
*J. fuscolineatus*	—	South Africa	Tugela Banks	JF493702	Steinke et al. 2016
*J. grypotus*	—	China	−	KC491206	Chao et al. 2019
*J. heterolepis*	NMMBA 34748	Malaysia	Bintulu	OK255556	Hanafi et al. 2024
*J. macropterus*	CSIRO H 7370-09	Indonesia	Palabuhanratu	JN312943	This study
*J. macropterus*	CSIRO H 7370-10	Indonesia	Palabuhanratu	JN312944	This study
*J. macropterus*	CSIRO H 7370-10	Indonesia	Palabuhanratu	JN312945	This study
*J. majan*	—	Dubai	−	KP722728	Lo et al. 2015
*J. novaeguineae*	—	Australia	Northern Territory	KX777978	Lo et al. 2017
*J. plagiostoma*	NMMBA 37101	Malaysia	Goebilt	OK255570	Hanafi et al. 2024
*J. sasakii*	NMMBA 37095	Malaysia	Sandakan	OK257864	Hanafi et al. 2024
*J. taiwanensis*	—	China	Dongshan island	MG917694	Chao et al. 2019
*J. trachycephalus*	—	Thailand	−	KX777979	Lo et al. 2017
*J. trewavasae*	—	China	Dongshan island	MG917694	Chao et al. 2019
*J. weberi*	NMMBA 37097	Malaysia	Beluran	OK392095	Hanafi et al. 2024
*Dendrophysa russelii*	—	China	−	JQ728562	Chao et al. 2019

## ﻿Taxonomy

### ﻿ Johnius javaensis

Taxon classificationAnimaliaAcanthuriformesSciaenidae

sp. nov. Lin, Pogonoski, Fahmi, Wibowo, Chao & Seymour, 2025

48F92633-7D63-5EF9-8CB9-4FF4249C2D28

https://zoobank.org/A80EC468-78F9-45F3-AC87-69CE6342C168

**Figure 2.**
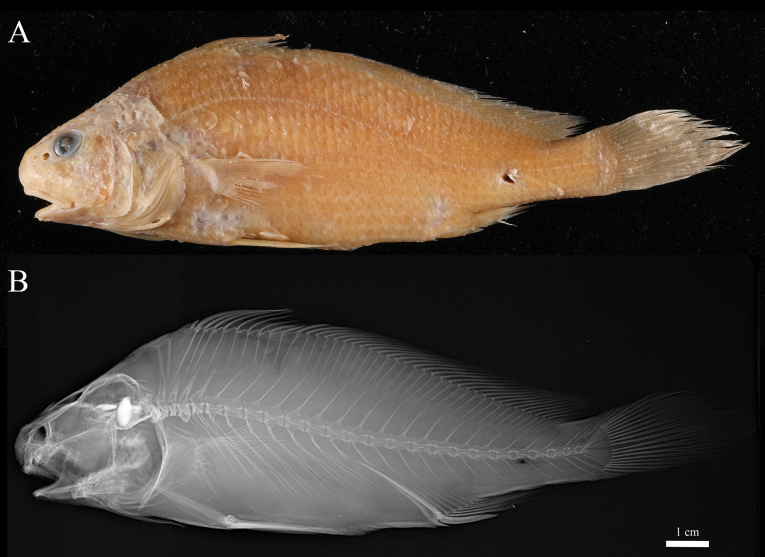
Holotype of *Johnius javaensis* sp. nov., MZB 28379, 121 mm SL, Cilacap, Central Java, Indonesia. A. Preserved specimen; B. Digital radiograph of preserved specimen.

**Figure 3.**
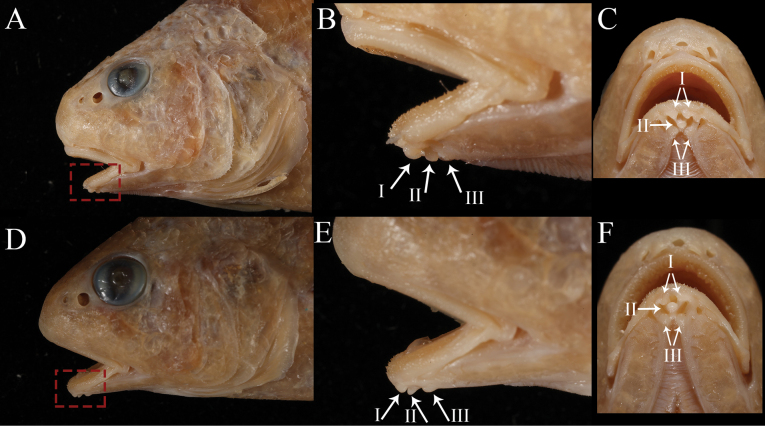
Five vesicular mental barbels of *Johnius javaensis* sp. nov. at chin. A, D, B, E. Lateral views; C, F. Ventral views; A–C. MZB 28379, holotype, 121 mm SL; D–F. CSIRO H 8376-12, paratype, 112 mm SL. (I) anterior pair of mental barbels, (II) middle mental barbel, (III) posterior pair of mental barbels. Not to scale.

**Figure 4.**
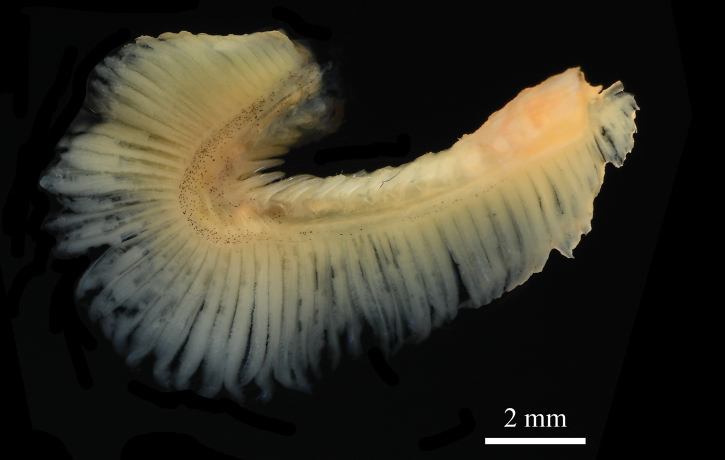
First gill arch on right side of *Johnius javaensis* sp. nov., CSIRO H 8376-12, paratype, 112 mm SL.

**Figure 5.**
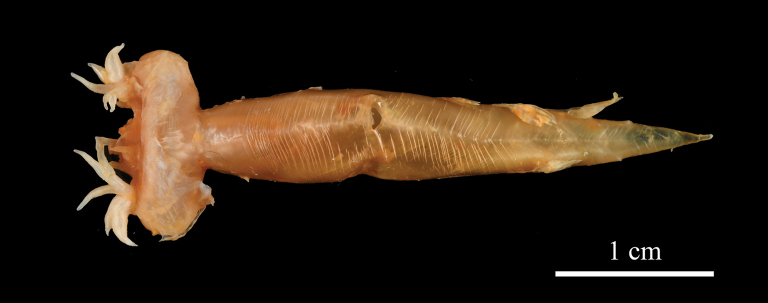
Swimbladder (ventral view) of *Johnius javaensis* sp. nov., CSIRO H 8376-12, paratype, 112 mm SL.

**Figure 6.**
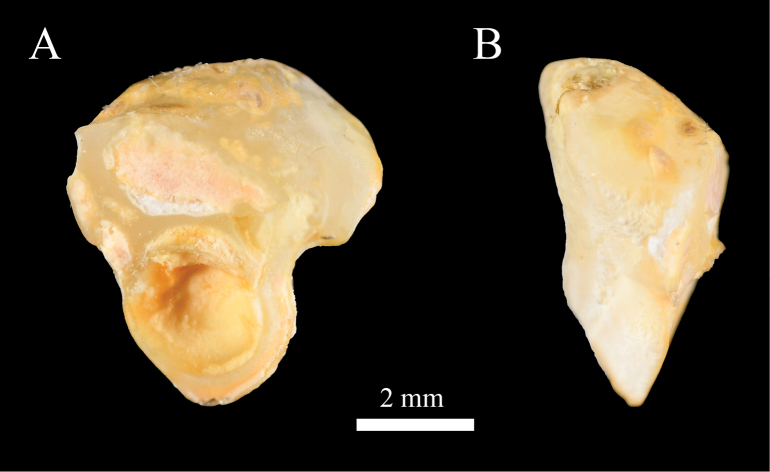
Right side of sagitta of *Johnius javaensis* sp. nov. A. Medial view; B. Lateral view, CSIRO H 8376-12, paratype, 112 mm SL.

**Figure 7.**
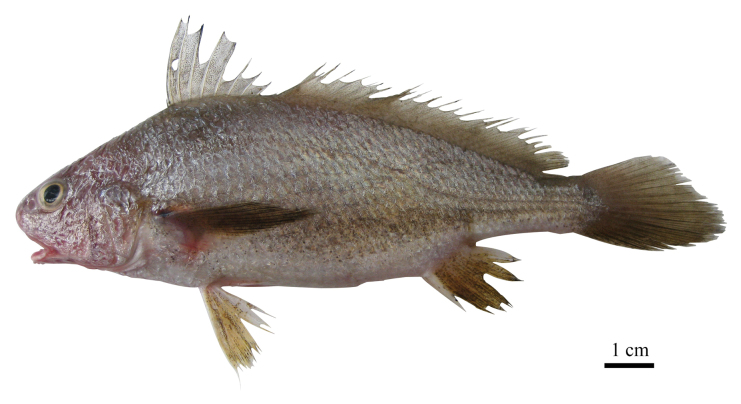
Fresh specimen of *Johnius javaensis* sp. nov. CSIRO H 7697-11, paratype, 115 mm SL, male, Cilacap, Central Java, Indonesia.

Figs 2, 3, 4, 5, 6, 7, Table 2 New English common name: Java croaker New Indonesian name: Gulamah Jawa

Johnius heterolepis (text, in part; figure incorrectly duplicated from Nibea soldado): White et al. (2013: 236) (Indonesia).

#### Type material.

***Holotype*.** • MZB 28379, 121 mm SL, female, Cilacap, Central Java, **Indonesia**, 19 October 2008. ***Paratypes*.** • CSIRO H 7697-11, 115 mm SL, male, collected with holotype; • CSIRO H 8376-12, 112 mm SL, Cilacap, Central Java, Indonesia (23 March 2010); • CSIRO H 9285-33, 114 mm SL, Cilacap, Central Java, Indonesia (01 July 2004); • MZB 28380, 87 mm SL, Palabuhanratu, West Java, Indonesia (19 March 2010).

#### Diagnosis.

A new species of *Johnius* characterized by five vesicular mental barbels on the chin, 11 or 12 swim-bladder appendages, and the ventral margin of the sagitta head expanded into a distinct convexity.

#### Description.

Dorsal fin X + I, 32 (X + I, 30–32); anal fin II, 7; pectoral fin rays 18 (17–18); caudal fin rays 17; pored lateral line scales 48 (46–48); scale rows above lateral line 5 (4–5), below 8 (7–8); outer gill rakers of 1^st^ arch 6 + 1 + 12 = 19 (5–6 + 1 + 11–12 = 17–19); vertebrae 11 + 14, last well-developed pleural rib on 10^th^ vertebra, first anal proximal radial between 11^th^–12^th^ vertebrae; swim-bladder appendages 11–12. Proportions as % of SL: head length 28.4 (25.9–27.2); eye diameter 5.4 (5.8–6.1); body depth 31.7 (27.1–29.2); body width 18.4 (14.5–16.7). Proportions as % of HL: eye diameter 18.9 (22.2–22.6); snout length 27.0 (24.4–25.5); interorbital width 26.2 (25.2–27.6); post-orbital length 58.1 (53.1–57.1); maxillary length 35.5 (34.2–35.9); second anal spine length 30.8 (34.5–40.1).

Body moderately deep, dorsal profile evenly arched, ventral profile rather flat; head conical, 25.9–28.4% of SL. Snout bluntly rounded, projecting in front of upper jaw; three upper and three marginal snout pores; underside of lower jaw with five mental pores, median mental pore with two small openings inside, very closely positioned. Five vesicular mental barbels at chin, with anterior pair of mental barbels on both sides of anterior point of inner mental pore, one middle mental barbel at the posterior end of median mental pore, and posterior pair of mental barbels at the posterior end of outer mental pore. Mouth inferior with upper jaw longer than lower jaw, upper jaw extending posteriorly to below middle of eye or hind margin of pupil. Upper jaw with a single, outer row of teeth slightly enlarged, and an inner band of small, conical teeth. Lower jaw with broad band of uniformly small, conical teeth. Eye circular, eye diameter small in holotype (18.9% of HL) and moderately large in paratypes (22.2–22.6% of HL). Eye diameter smaller than interorbital width. Anterior and posterior nostrils circular and somewhat ovate, respectively, just anterior to eye; posterior nostril twice the size of the anterior. Gill rakers short and stiff, its length approximately 31.4–46.2% of gill filament length at angle of the gill arch. Scales large, cycloid on head (except operculum), throat, membrane of dorsal, anal and caudal fins; body scales ctenoid, ctenii well developed. Opercular scales a mix of cycloid and ctenoid. Third or fourth dorsal spine longest. First soft ray of pelvic fin with short filament. Second anal-fin spine stiff, its length 59.6–74.9% of first anal-fin ray and 30.8−40.1% of head length. Caudal fin rhomboid. Sagitta thick and triangular; ostium of sulcus in head with long axis lying obliquely to sagitta; cauda expanded and deepened as hollow cone; outer surface with crest-like elevations; ventral margin of sagitta head expanded to a distinct convexity. Swimbladder hammer-shaped, with 11 or 12 pairs of arborescent diverticula attached to anterior and lateral sides of main chamber, becoming tubular posteriorly. First anterior pair of diverticulae pierces through septum transversum, reaching to the base of cranium (basioccipital and exoccipital bones). Drumming muscles restricted to male (absent in female) and positioned on the lateral body cavity wall.

**Table 2.** Morphometrics and meristics of *Johnius javaensis* sp. nov.

	Holotype	Paratypes (n = 4)
**Morphometrics (mm)**		
Total length	150.0	110.0−143.0
Standard length	121.0	87.0−115.0
Head length	34.4	23.0−31.0
Eye diameter	6.5	5.1−7.0
Snout length	9.3	5.7−7.9
Interorbital width	9.0	5.8−8.4
Post-orbital length	20.0	12.8−17.7
Maxillary length	12.2	8.2−10.7
Upper jaw length	10.3	6.3−9.5
Lower Jaw length	8.3	5.5−8.1
Body depth (D1–P2)	38.4	30.3−33.6
Body width (P1–P1)	22.2	16.5−19.2
Second anal-fin spine length	10.6	10.0−12.5
First anal-fin ray length	17.8	15.1−17.0
Caudal-peduncle length	29.7	18.1−26.8
Caudal-peduncle depth	11.3	6.8−10.6
**Meristics**		
Dorsal fin	X+I, 32	X+I, 30−32
Anal fin	II, 7	II, 7
Pectoral rays	18	17−18
Outer gill rakers of 1^st^ arch	6+1+12	5−6+1+11−12
Pored lateral line scales	48	46−48
Scale rows above lateral line	5	4−5
Scale rows below lateral line	8	7−8
Vertebrae	11+14	11+14
Swimbladder appendages	11	12 (*n* = 1)
**Proportions as % SL**		
Head length	28.4	25.9−27.2
Eye diameter	5.4	5.8−6.1
Body depth	31.7	27.1−29.2
Body width	18.4	14.5−16.7
**Proportions as % HL**		
Eye diameter	18.9	22.2−22.6
Snout length	27.0	24.4−25.5
Interorbital width	26.2	25.2−27.6
Post-orbital length	58.1	53.1−57.1
Maxillary length	35.5	34.2−35.9
Second anal-fin spine length	30.8	34.5−40.1
**Proportions (%)**		
Eye diameter / interorbital width	72.2	81.3–87.9
Length lower jaw / upper jaw length	80.6	80.0–87.3
Gill raker / filament length	31.4	36.7–46.2
Caudal-peduncle depth / length	38.1	37.6–40.0
Second anal-fin spine / first anal-fin ray length	59.6	62.5–74.9

#### Coloration (based on color photographs of paratypes when fresh).

Body greyish dorsally; upper two-thirds of body darker, separated from whitish to greyish belly by a distinct line. Line darkish to greyish, with darkish dots from base of pectoral fins to base of caudal fin and essentially parallel to pectoral fin. Dorsal fin dusky, with small dots; pectoral-fin darkish to greyish, with one black dot on upper end of axil; pelvic fins yellowish, with sparse, small darkish dots; anal fin yellowish, with dense, small darkish dots; caudal fins darkish. Color in preservative (all type material): head, body and fins brownish tan; first dorsal and anal fins with dense, small darkish dots.

#### Geographical distribution.

All specimens of the new species were collected from the west and central coast of southern Java, Indonesia.

#### Etymology.

The specific name ‘‘*javaensis*’’ is proposed, as all collected specimens were from the west and central of southern Java.

#### Comparisons.

**Figure 8.**
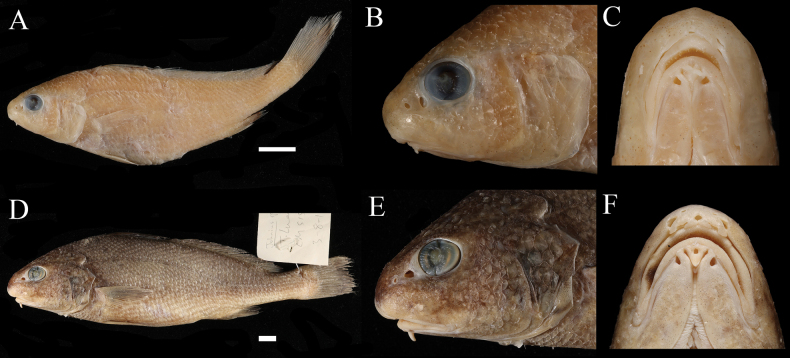
Comparison of the single short mental barbel in *Johnius macropterus* and *J. amblycephalus.* A–C. *J. macropterus*, CSIRO H 7370-09, 85 mm SL; D–E. *J. amblycephalus*, CSIRO H 7245-05, 182 mm SL; A, B, D, E. Lateral views; C, F. Ventral views. Scale bars: 1 cm (A, D); others not to scale.

The new species belongs to the subgenus Johnius (Johnius), which exhibits uniformly-sized lower jaw teeth or the inner rows of lower jaw teeth molariform. The Southeast Asia region, including Java, harbors three species that possess mental barbels, *J. javaensis* sp. nov., *J. macropterus*, and *J. amblycephalus*. The new species can be readily separated from *J. macropterus* and *J. amblycephalus* in having five vesicular mental barbels on the chin, while the other two species only have one mental barbel (Fig. 8; Table 3). Additionally, the new species can also be readily distinguished from similar congeners without barbels in the region (Table 3). *Johnius javaensis* sp. nov. can be distinguished from *J. heterolepis* in having more dorsal-fin rays (30–32 vs 25–28) and fewer swim-bladder appendages (11−12 vs 14). The new species can be separated from *J. hypostoma* in having fewer scale rows below the lateral line (7 or 8 vs 9 or 10), more swim-bladder appendages (11 or 12 vs 9–10), and a smaller interorbital width (25.2–27.6% vs 28.0–34.0% of head length). Furthermore, *Johnius javaensis* sp. nov. can be differentiated from *J. laevis* Sasaki & Kailola, 1991 in having body scales with well-developed ctenii. The new species can be distinguished from *J. macrorhynus* (Lal Mohan, 1976) in having fewer scale rows below the lateral line (7 or 8 vs 10–14), more lower gill rakers (11 or 12 vs 5–8), and fewer swim-bladder appendages (11 or 12 vs 13–16) (Table 3).

**Table 3.** Selected diagnostic characters of *Johnius javaensis* sp. nov. and related species.

Species	J. javaensis sp. nov.	J. macropterus	J. amblycephalus	J. heterolepis	J. hypostoma	J. laevis	J. macrorhynus
Mental barbel	5	1	1	0	0	0	0
Dorsal-fin rays	30–32	28–33	23–26	25–28	31–33	29–34	25–30
Anal-fin rays	7	7	7	7	7–8	7	7–8
Scale rows above lateral line	4–5	4–7	7–12	4–5	5–6	5–6	5–9
Scale rows below lateral line	7–8	9–12	13–18	8–11	9–10	8–10	10–14
Gill rakers	5–6+1+11–12	4–5+1+7–12	4+1+6–9	4–5+1+9–12	11–12 (lower)	5–7+1+10–12	3–5+1+5–8
Vertebrate (pleural rib present + absent)	24 (11+14)	25 (11+14)	25	25 (10+15)	25 (11+14 or 12–13)	25 (10+15)	25
Swimbladder appendages	11–12	14–15	14–15	14	9–10	11–14	13–16
HL (% SL)	25.9–28.4	24.1–27.6	29.9–31.8	25.0–32.7	28.3–28.6	29.9–33.8	26.0–31.4
Eye diameter (% HL)	18.9–22.6	18.0–26.0	19.0–23.4	19.0–27.0	19.0–23.0	22.4–30.0	19.0–27.0
Interorbital width (% HL)	25.2–27.6	24.9–27.6	27.5–29.3	20.3–28.2	28.0–34.0	24.6–29.8	21.6–25.7
Ctenii on body scales	Well developed	Well developed	Cycloid	Well developed	Well developed	Poorly developed	Well developed
Distribution	Indonesia	India to Papua New Guinea	Pakistan to China and Australia	Malaysia to Indonesia	Malaysia to Indonesia	Australia and Papua New Guinea	Pakistan to Indonesia
References	This study	Trewavas 1977; Sasaki 2001; this study	Sasaki 2001; Hanafi et al. 2023	Sasaki 1992; Hanafi et al. 2022	Sasaki and Kailola 1991; Sasaki 2001	Sasaki and Kailola 1991	Lal Mohan 1975; Trewavas 1971; Sasaki 2001

#### Phylogeny of *Johnius javaensis* sp. nov.

Based on the ML tree analysis, the *Johnius* species with mental barbels are non-monophyletic and merit further investigation. *Johnius javaensis* sp. nov. is sister to *J. macropterus* with high bootstrap values (Fig. 9). The intraspecific K2P distance in the 521-bp COI gene sequences for the new species are 1.83% (0.77−2.56%) (Table 4). The K2P distance between *J. javaensis* sp. nov. and *J. macropterus* is 8.18% (7.76−8.84%), which is lower than the distance between *J. javaensis* sp. nov. and the other 19 *Johnius* species in this study, averaging 19.01% (12.68%−30.53%), giving high confidence in species relationships (Table 4). Our analysis reveals substantial interspecific divergences among the 21 *Johnius* species, with an average K2P distance of 18.78% ± 5.79% (range: 1.96%–32.24%). Notably, the exceptionally low genetic distance (< 2%) between *J. taiwanensis* Chao, Chang, Chen, Guo, Lin, Liou, Shen & Liu, 2019 and *Johnius sasakii* Hanafi, Chen, Seah, Chang, Liu & Chao, 2022 suggests potential taxonomic ambiguity and warrants further investigation (Table 4).

**Figure 9.** Phylogenetic relationship of the new species *Johnius javaensis* sp. nov. and 20 *Johnius* species from the Indo-West Pacific region and one sciaenid as the outgroup based on maximum-likelihood (ML) analyses. Nodes are supported by bootstrap values of distance matrix. Only values ≥50% are presented.

**Table 4.** Genetic distances (Kimura 2-parameter distance, %) within species and between species of *Johnius*.

	1	2	3	4	5	6	7	8	9	10	11	12	13	14	15	16	17	18	19	20
**1. *Johnius javaensis* sp. nov.**	**1.82 (0.77–2.56)**																			
2. *Johnius macropterus*	8.18																			
3. *Johnius amblycephalus*	29.69	26.79																		
4. *Johnius belangerii*	16.89	14.80	29.07																	
5. *Johnius borneensis*	17.91	17.85	27.05	15.65																
6. *Johnius carouna*	17.84	15.15	26.74	15.99	18.25															
7. *Johnius coitor*	18.15	16.96	26.09	16.48	18.47	15.34														
8. *Johnius distinctus*	18.69	15.86	25.24	17.39	10.88	19.19	18.18													
9. *Johnius dussumieri*	18.78	18.07	22.03	17.63	10.93	18.42	18.21	9.98												
10. *Johnius elongatus*	29.08	27.01	19.02	27.85	30.29	28.26	28.43	27.42	27.62											
11. *Johnius fuscolineatus*	28.72	26.36	9.39	28.70	27.10	25.06	24.84	25.29	22.07	18.51										
12. *Johnius grypotus*	14.05	14.76	29.64	16.61	15.93	14.99	15.07	16.10	15.84	31.96	25.69									
13. *Johnius heterolepis*	15.44	13.34	25.96	13.41	14.49	13.09	12.29	15.39	14.73	28.92	24.14	11.95								
14. *Johnius majan*	19.17	17.59	21.67	18.44	12.75	19.26	18.51	11.52	9.34	25.58	21.99	18.40	16.01							
15. *Johnius novaeguineae*	13.03	11.25	28.25	12.92	16.18	14.93	14.90	15.86	14.70	29.24	24.59	11.53	9.97	15.47						
16. *Johnius plagiostoma*	20.46	17.75	30.99	18.57	20.52	20.21	21.51	19.29	20.29	32.24	29.64	22.12	19.09	20.82	20.82					
17. *Johnius sasakii*	15.54	13.10	27.31	15.80	17.34	17.90	16.12	16.85	17.12	28.61	27.58	15.22	13.65	18.40	13.69	19.69				
18. *Johnius trachycephalus*	16.61	15.21	30.01	14.40	13.47	16.43	18.33	15.35	16.82	31.68	28.47	13.32	13.56	17.07	12.17	18.58	14.29			
19. *Johnius taiwanensis*	16.54	13.58	27.01	14.80	16.59	16.88	15.61	17.36	17.12	28.61	26.97	15.22	13.41	17.63	13.44	20.23	1.96	13.56		
20. *Johnius trewavasae*	15.30	12.84	28.34	14.31	16.40	15.12	19.01	17.87	18.62	30.42	26.97	16.26	14.31	18.92	13.13	18.00	14.84	14.99	13.61	
21. *Johnius weberi*	16.90	13.35	26.92	13.71	15.74	17.75	18.17	15.65	16.45	27.42	24.76	14.36	13.95	17.00	11.35	21.64	12.91	12.18	12.43	14.82

#### Remarks.

*Johnius javaensis* sp. nov. is a small sciaenid; the holotype (121.0 mm SL) is mature, with fully developed gonads. The specimens were collected from coastal fish markets, but the specific habitat preferences of this species are unknown.

### ﻿Key to the species of *Johnius* around the Java Island

**Table:** 

1	Mouth terminal to subterminal; inner row of teeth on lower jaw more or less enlarged and spaced; outer teeth of upper jaw widely spaced	**2**
–	Mouth inferior; lower jaw teeth uniformly sized or a few rear inner teeth molariform; outer teeth of upper jaw not widely spaced	**3**
2	First pair of mental pores separated by symphysis, snout rounded; second anal-fin spine length 40–50% of head length	***J. plagiostoma* (Bleeker, 1850)**
–	First pair of mental pores by a crescent-shaped groove, snout pointed; second anal-fin spine length 24–42% of head length	***J. borneensis* (Bleeker, 1850)**
3	Chin with barbel	**4**
–	Chin without barbel	**6**
4	Chin with single barbel	**5**
–	Chin with five vesicular barbels	***J. javaensis* sp. nov.**
5	Scales cycloid on head and body	***J. amblycephalus* (Bleeker, 1855)**
–	Scales ctenoid on head and body	***J. macropterus* (Bleeker, 1853)**
6	Lower jaw with inner row of molariform teeth posteriorly	***J. macrorhynus* (Mohan, 1976)**
–	Lower jaw teeth all conical, uniform in size	**7**
7	Scale rows above lateral line to origin of dorsal fin 4 or 6; scale rows below lateral line to origin of anal fin 8–11	**8**
–	Scale rows above lateral line to origin of dorsal fin 7 to 11; scale rows below lateral line to origin of anal fin 11–17	**10**
8	Dorsal-fin rays 31–33	***J. hypostoma* (Bleeker, 1853)**
–	Dorsal-fin rays 24–30	**9**
9	Second anal-fin spine length 41 to 58% of head length	***J. carouna* (Cuvier, 1830)**
–	Second anal-fin spine length 25 to 34% of head length	***J. heterolepis* Bleeker, 1873**
10	Body darkly pigmented; lower fins black	***J. belangerii* (Cuvier, 1830)**
–	Body not darkly pigmented; lower fins pale	**11**
11	Snout slightly projecting; first gill arch with 7–10 gill rakers on lower limb	***J. latifrons* Sasaki, 1992**
–	Snout bluntly rounded; first gill arch with 9–12 gill rakers on lower limb	***J. carouna* (Cuvier, 1830)**

### ﻿Additional material examined

*Johnius macropterus*: CSIRO H 7370-09, 85 mm SL, CSIRO H 7370-10, 79–85 mm SL (2 specimens), Palabuhanratu, West Java, Indonesia (11 August 2010), CSIRO H 7250-18, 113 mm SL, Palabuhanratu, West Java, Indonesia (19 March 2010).

*Johnius amblycephalus*, CSIRO H 7245-05, 182 mm SL, Tanjung Luar, Lombok, Indonesia (03 August 2010).

## Supplementary Material

XML Treatment for Johnius javaensis

## ﻿Additional information

### Use of AI

No use of AI was reported.

### Author ORCIDs

Bai-an Lin https://orcid.org/0000-0002-5966-9062

John J Pogonoski https://orcid.org/0000-0002-4878-3919

Fahmi https://orcid.org/0000-0003-3199-7467

Kunto Wibowo https://orcid.org/0000-0003-4465-3022

Ning Labbish Chao https://orcid.org/0000-0002-0474-8740

Mathew Seymour https://orcid.org/0000-0002-2441-9629

### Data availability

All data that support the findings of this study are available in the main text. All sequences generated and analyzed in this study are available in GenBank and BOLD with accession number and references in Table 1.
